# Evaluation of Estrogenic Potential of Flavonoids Using a Recombinant Yeast Strain and MCF7/BUS Cell Proliferation Assay

**DOI:** 10.1371/journal.pone.0074881

**Published:** 2013-10-01

**Authors:** Flávia A. Resende, Ana Paula S. de Oliveira, Mariana S. de Camargo, Wagner Vilegas, Eliana A. Varanda

**Affiliations:** 1 Department of Biological Sciences, Faculty of Pharmaceutical Sciences, Univ Estadual Paulista, Araraquara, São Paulo, Brazil; 2 Campus do Litoral Paulista-Unidade São Vicente, Univ Estadual Paulista, São Vicente, São Paulo, Brazil; Michigan State University, United States of America

## Abstract

Phytoestrogens are of interest because of their reported beneficial effects on many human maladies including cancer, neurodegeneration, cardiovascular disease and diabetes. Furthermore, there is a search for compounds with estrogenic activity that can replace estrogen in hormone replacement therapy during menopause, without the undesirable effects of estrogen, such as the elevation of breast cancer occurrence. Thus, the principal objective of this study was to assess the estrogenic activity of flavonoids with different hydroxylation patterns: quercetin, kaempferol, luteolin, fisetin, chrysin, galangin, flavone, 3-hydroxyflavone, 5-hydroxyflavone and 7-hydroxyflavone via two different *in vitro* assays, the recombinant yeast assay (RYA) and the MCF-7 proliferation assay (*E-screen*), since the most potent phytoestrogens are members of the flavonoid family. In these assays, kaempferol was the only compound that showed ERα-dependent transcriptional activation activity by RYA, showing 6.74±1.7 nM EEQ, besides acting as a full agonist for the stimulation of proliferation of MCF-7/BUS cells. The other compounds did not show detectable levels of interaction with ER under the conditions used in the RYA. However, in the *E-screen* assay, compounds such as galangin, luteolin and fisetin also stimulated the proliferation of MCF-7/BUS cells, acting as partial agonists. In the evaluation of antiestrogenicity, the compounds quercetin, chrysin and 3-hydroxyflavone significantly inhibited the cell proliferation induced by 17-β-estradiol in the *E-screen* assay, indicating that these compounds may act as estrogen receptor antagonists. Overall, it became clear in the assay results that the estrogenic activity of flavonoids was affected by small structural differences such as the number of hydroxyl groups, especially those on the B ring of the flavonoid.

## Introduction

An important class of toxicity arising from the environment is endocrine disruption. This is an especially harmful effect of chemical pollution that occurs when a chemical accumulates in an animal (or human) and affects its endocrine system. Given the multiple functions of hormones in the body, this alteration can lead to a variety of adverse effects, including hermaphroditism in fish and reproductive deficiencies in humans [1], [2].

On the other hand, compounds that either induce or inhibit cellular estrogen responses have potential value as biochemical tools and candidates for drug development. Since the discovery of the nonsteroidal estrogens, many estrogen agonists and antagonists have been developed as agents for regulating fertility, preventing and controlling hormone-responsive breast cancer, and post-menopausal hormone replacement [3].

Thus, estrogenicity of flavonoids has become an important issue, since the most potent phytoestrogens are members of the flavonoid family. Besides their estrogenic properties, phytoestrogens exert a wide variety of pharmacological effects in animal cells, including inhibition of tyrosine kinases and DNA topoisomerases, antioxidative effects, interference in a plethora of signaling pathways, cell cycle, and apoptosis events, synergism with growth factors by inducing synthesis or activating receptors, and modulation of important enzymatic activities [4].

*E-screen* determines the (anti) estrogenicity of compounds indirectly through measurement of the proliferation of MCF-7 cells. The rationality for the use of this cell line was: (1) it is a cell line of endocrine origin and is ER positive; (2) expresses aromatase and 5α-reductase like other steroidogenic cells; (3) possesses endogenous aromatase activity which converts androgens to estrogens and (4) can elicit an estrogen-induced response involving both genomic and non-genomic pathways. Therefore by using MCF-7 cells it is always possible to link the (anti) estrogenic potential of flavonoids with its carcinogenic activity, if there is any. This information is critical since there are several reports suggesting the fact that endocrine disrupting chemicals directly or indirectly promote the rising incidence of breast, prostate and testicular cancer, cryptorchidism, ectopic pregnancies, and early endometriosis. However, one of the limitations for determining estrogenicity of chemicals by checking the proliferation of ER positive MCF-7 cell line is that mitogens other than estrogens can also influence cell proliferation thus rendering non-specific responses by chemicals [5].

Thus, to complement the assay of proliferation of MCF-7 cells, we included the recombinant yeast-based estrogenicity assay, also known as recombinant yeast assay (RYA). RYA utilizes an engineered yeast strain in which the transcription of a reporter gene depends upon the presence in the medium of compounds capable of binding to ERα [6]. This is a simplified version of the mechanism by which natural estrogens operate in vertebrates; the fundamental similarity of transcription in all eukaryotes ensures that it also works in yeast [7].

Two characteristics of the yeast cell contribute to the success of the RYA. First, yeast has no endogenous system homologous to vertebrate nuclear receptors that could interfere with the assay. Second, the folding and post translational processing of vertebrate protein in yeast is very similar to the one from mammalian cells, which results in the preservation of the native receptor structure when expressed in yeast. This is of paramount interest for our purposes, since the correct structure of the ligand-binding domain of the receptor determines the specificity of the system, that is, its capability to distinguish between ligands and non-ligands. This latter point has been tested in numerous reports comparing ligand activity of different compounds in yeast and in mammalian systems. Although some differences do occur, RYA always stands as a reliable method to detect and characterize vertebrate receptor ligands [8].

**Figure 1:**
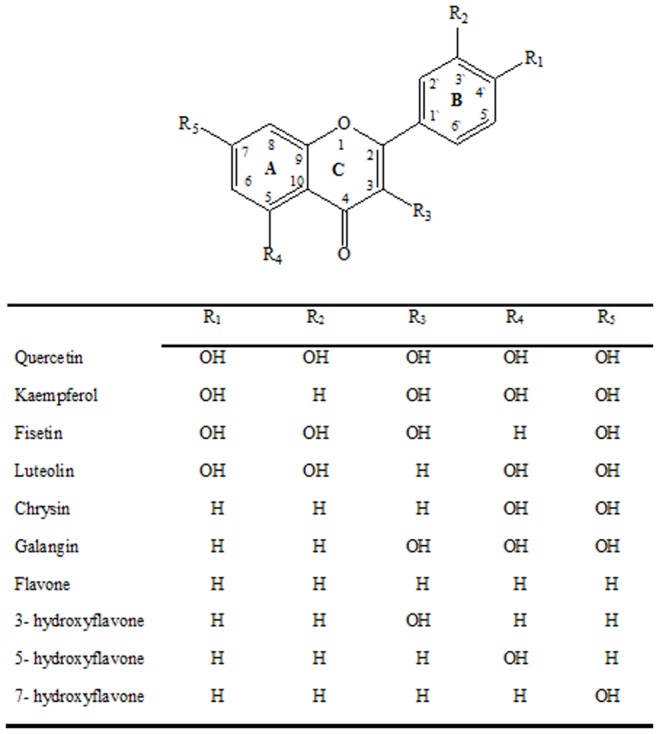
Molecular skeleton of flavonoids.

In this work we investigated the potential estrogenicity of ten flavonoids (quercetin, kaempferol, luteolin, fisetin, galangin, chrysin, flavone, 3-hydroxyflavone, 5-hydroxyflavone and 7-hydroxyflavone) with different hydroxylation patterns (**Figure 1**) by combining two *in vitro* methods that monitor the estrogen responses that occur in vertebrate cells.

The two assays combined allow the analyst to estimate the affinity of a series of compounds for the human estrogen receptor (ER), as well as to explore the possible physiological consequences of this interaction [9].

## Materials and Methods

### Chemicals and Culture Media

Quercetin, kaempferol, fisetin, luteolin, flavone, 3-hydroxyflavone, 5-hydroxyflavone, 7-hydroxyflavone, chrysin, galangin and dimethyl sulfoxide (DMSO) were purchased from Sigma Chemical Co. (St. Louis, MO, USA). Yeast was grown on complete medium (10 g/L Bacto™ yeast extract, 20 g/L Bacto™ peptone, BD, Sparks, Maryland, USA; 20 g/L glucose) and minimal medium (6.7 g/L yeast nitrogen base without amino acids, Difco, Basel, Switzerland, 20 g/L glucose, supplemented with 0.1 g/L of prototrophic markers as required). The human MCF-7/BUS breast cancer cells were cultured in Dulbecco’s Modified Eagle Medium (DMEM) with 15 mg/L phenol red, 10% fetal bovine serum (FBS), 2% of 200 mM L-glutamine, 2% of 1 M HEPES buffer, 1% of 100 mM sodium pyruvate and 1% of 10 mg/mL penicillin–streptomycin, at 37°C, in an atmosphere of 5% CO_2_ and 95% air at saturating humidity.

### Recombinant Yeast Assay (RYA)

The RYA tests were performed essentially as in Garcia-Reyero et al. [6]. Briefly, yeast strain BY4741 (MATa ura3Δ0 leu2Δ0 his3Δ1 met15Δ0) (EUROSCARF, Frankfurt, Germany), which was kindly provided by Dr Benjamin Piña (CSIC, Barcelona, Spain), was transformed with plasmids pH5HE0 and pVitBX2 [6].

Expression plasmid pH5HE0 contains the human estrogen hormone receptor gene HE0 [10], cloned into the constitutive yeast expression vector pAAH5 [11]. The reporter plasmid pVITB2x contains two copies of the pseudo-palindromic estrogen responsive element from the *Xenopus laevis* vitellogenin B1 gene (5′-AGTCACTGTGACC-3′) inserted into the unique KpnI site of pSFLΔ-178K [9].

Transformed clones were first grown in 3 mL of rich complete medium at 30°C. Next, they were grown overnight in minimal medium. The final culture was adjusted to an optical density (OD) of 0.1 at 600 nm and distributed in the wells of a siliconized 96-well polypropylene microliter plate (NUNC™, U96 PP 0.5 mL), at 90 µL in the first row. Aliquots of 10 µL of the flavonoids, at initial concentration of 0.125 g/L, were dispensed into wells on the first row and serial dilutions were prepared along the plate, containing flavonoid with dilution factors 1∶10, 1∶30, 1∶90, 1∶270 and 1∶810.

A positive control was made by adding 17-β-estradiol at a final concentration of 10 nM. Moreover, we included a toxicity control, by adding 10 nM of 17-β-estradiol to a sample with a dilution factor of 1∶30, and 10% DMSO as the negative control.

Plates were incubated for 6 h at 30°C under mild shaking. After incubation, 50 µL of Y-PERTM (PIERCE™, Rockford, IL, USA) was added to each well and incubated at 30°C for a further 30 min. Afterwards, 50 µL of assay buffer was added to the lysed cells. The assay buffer was prepared by mixing 100 mL Z-buffer, 1 mL Triton X-100 (Sigma), 1 mL SDS 10%, 70 µL 2-mercaptoethanol (Fluka) and 21 mg of 4-methylumbelliferyl β-D-galactoside (Sigma). Z-Buffer is a mix of: 60 mM Na_2_HPO_4_, 40 mM NaH_2_PO_4_, 10 mM KCl and 1 mM MgSO_4_, pH 7.0.

After centrifugation, plates were read in a spectrofluorometer (Synergy H1, Biotek), at 355 nm excitation and 460 nm emission wavelengths. Fluorescence was recorded for 20 min (one measurement per min); β-galactosidase activity was calculated as the rate of increase of fluorescence (in arbitrary units). Data analysis of fluorescence units was performed with GraphPad Prism 5.0 (GraphPad Software Inc., San Diego, CA), using the ANOVA test followed by the Tukey test (*p*<0.05). RYA does not provide a direct measurement of the molar (or mass) concentration of endocrine disruptors, but of their estrogenic activity. For simplification, results were calculated as estradiol equivalents (EEQ), defined as the amount of estradiol that should be present to account for the observed response in a given sample. These equivalents were calculated from the lowest dilution in which the *β*-galactosidase activity was indistinguishable from that of the control (only vehicle).

To translate results from serial dilutions to EEQ, we assumed that hormonal dose-response curves follow a sigmoidal function:
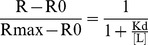
in which R0, R, and Rmax represent *β*-galactosidase units obtained without ligand (or extract) addition, at a given ligand concentration [L], and at a saturating ligand concentration, respectively. Kd represents the dissociation constant of the ligand–hormone complex; its value coincides with EC_50_, the ligand concentration giving 50% of the maximal response. For extract serial dilutions, plotting dilution factors versus relative response followed an inverse sigmoidal function, in which the apparent EC_50_ correspond to the dilution (actual or theoretical) giving 50% the response for 10 nM estradiol. Apparent EC_50_ values for each sample (a minimum of two replicas with at least four points each) were calculated using standard non-linear regression methods These values were converted to EEQ by assuming they correspond to the EC_50_ of estradiol [5]–[9], 72.9±28 pM in our assay. Six independent experiments were done with the flavonoids, all of them in triplicate.

### E-screen Assay

The simple and sensitive *E-screen* cell proliferation assay was performed with the human MCF-7/BUS breast cancer cell line. MCF-7/BUS cells were used according to a technique slightly modified Villalobos et al. [12] from that originally described Soto et al. [13]. As an established estrogenic cell line these cells endogenously express ERα. In absence of any other reporter constructs they therefore allow a reliable detection of potential transcriptional changes caused by xenoestrogens [14].

Human MCF-7/BUS breast cancer cells were kindly provided by the Laboratory of Medical Investigations (Department of Environmental Medicine; University of Granada, Granada, Spain), and were cultivated in DMEM with phenol red, supplemented with 10% FBS at 37°C in an atmosphere of 5% CO_2_ and 95% air under saturating humidity.

Growth stimulation of MCF-7/BUS by compounds was measured as described in Soto et al. [13], with modifications by Villalobos et al. [12].

Subconfluent MCF-7/BUS cells were trypsinized and seeded in 24-well plates to an initial concentration of 20,000 cells per well in DMEM with 10% (v/v) FBS (1 mL/well). After cell adhesion to well bottoms (24 h incubation; 37°C, 5% CO_2_), the cells were washed with phosphate-buffered saline (PBS) and the culture medium was changed to DMEM supplemented with 10% charcoal dextran-stripped (steroid-free) FBS. The steroid-free experimental medium consisted of phenol-red- free DMEM supplemented with 10% stripped FBS, 2% of 200 mM L-glutamine, 2% of 1 M HEPES buffer, 1% of 100 mM sodium pyruvate and 1% of 10 mg/mL penicillin-streptomycin.

Positive and negative controls were 1×10^−8^ M 17-β-estradiol and steroid-free experimental medium, respectively. There was also a solvent control (DMSO at 0.01%, the maximum concentration of solvent used in the test) and medium control (10% FBS in DMEM).

Test compounds were added to experimental medium at a range of concentrations from 1×10^−9^ to 1×10^−5^ M. The concentrations were selected on the basis of a preliminary toxicity test based on sulforhodamine B (SRB) assay. Each experiment was performed three times on triplicate samples. The assay was terminated after 144 h of incubation by removing the medium from the wells and then the SRB assay was carried out.

For antiestrogenicity tests, before incubation, 1×10^−8^ M of 17-β-estradiol was added to the wells.

The estrogenic activity results were expressed as mean ± standard deviation of the proliferative effect (PE), which represents the maximum proliferation induced by the flavonoids in the MCF-7/BUS cells. This parameter was calculated according to Schiliró et al. [15], and is the ratio between the highest cell number achieved with the sample or 17-β-estradiol and the cell number in the solvent control (0.01% DMSO):
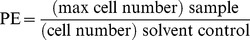



The estrogenic activity of a sample is evaluated by determining the relative efficacy of stimulation, called the relative proliferative effect (RPE%). The RPE compares the maximum proliferation induced by a sample with that induced by 17-β-estradiol:
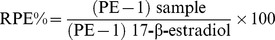



The RPE can be used to define full agonists for ER, between 80% and 100% relative proliferation. Partial and weak agonists induce a relative cell proliferation from 25% up to 80%, or 10% to 25%, respectively [16].

Formulas and functions of Excel (Microsoft, NY, USA) were used to calculate these results. Moreover, data analysis of estrogenic activity was performed with GraphPad Prism 5.0 (GraphPad Software Inc., San Diego, CA), using the ANOVA test followed by the Tukey test. Data analysis of antiestrogenic activity also was performed with GraphPad Prism 5.0 (GraphPad Software Inc., San Diego, CA), using the ANOVA test, however followed by the Dunnet test, to detect significant inhibition of MCF-7 proliferation after flavonoid treatment. Three independent experiments were done with the flavonoids, all of them in triplicate.

### Cytotoxicity Assay

The viability of the cells was measured with the SRB assay. The cells (2×10^3^ cells/well) were added to 96-well plates and the culture was maintained at 37°C under 5% CO_2_. The cells were attached for 24 h, and test compounds (17-β-estradiol and flavonoids) were added to steroid-free experimental medium, at a range of concentrations from 1×10^−9^ to 1×10^−3^ M. The assay was terminated after 6 days by removing media from the well and then the SRB assay was performed. For this assay, the cells were washed twice with ice-cold PBS before being fixed with 10% TCA (100 µL per well) at 4°C for 30 min. Each well was washed with tap water 5–6 times and dried thoroughly. 0.4% SRB dissolved in 1% acetic acid was added and incubated for 30 min. The wells were washed 5–6 times with 1% acetic acid and dried. To each dried well, 10 mM Trizma base (pH 10.5) was added. Optical density was measured at 530 nm in a microplate reader (Synergy H1, Biotek).

## Results

A total of 10 flavonoids were analyzed by the RYA assay. Among them, only kaempferol showed significant estrogenic activity, with ERα-dependent transcriptional activation activity, showing EC_50_ of 17.4±3.2 µM and 6.74±1.7 nM EEQ (**Table 1**). The other compounds (quercetin, fisetin, chrysin, luteolin, galangin, flavone, 3-hydroxyflavone, 5-hydroxyflavone and 7-hydroxyflavone) showed no detectable levels of estrogenicity.

**Table 1:** Estradiol equivalents (EEQ) and median effective concentration (EC_50_) of the kaempferol from the recombinant yeast assay.

Compounds	EEQ^a^	EC_50_ b
Kaempferol	6.74±1.7 nM	17.4±3.2 µM
Estradiol	−	72.9±28 pM

a EEQ (estradiol equivalents) = concentration of estradiol that elicit the same response as the sample in the RYA assay.

b EC_50_ = the ligand concentration giving 50% of the maximal response.

**Figure 2:**
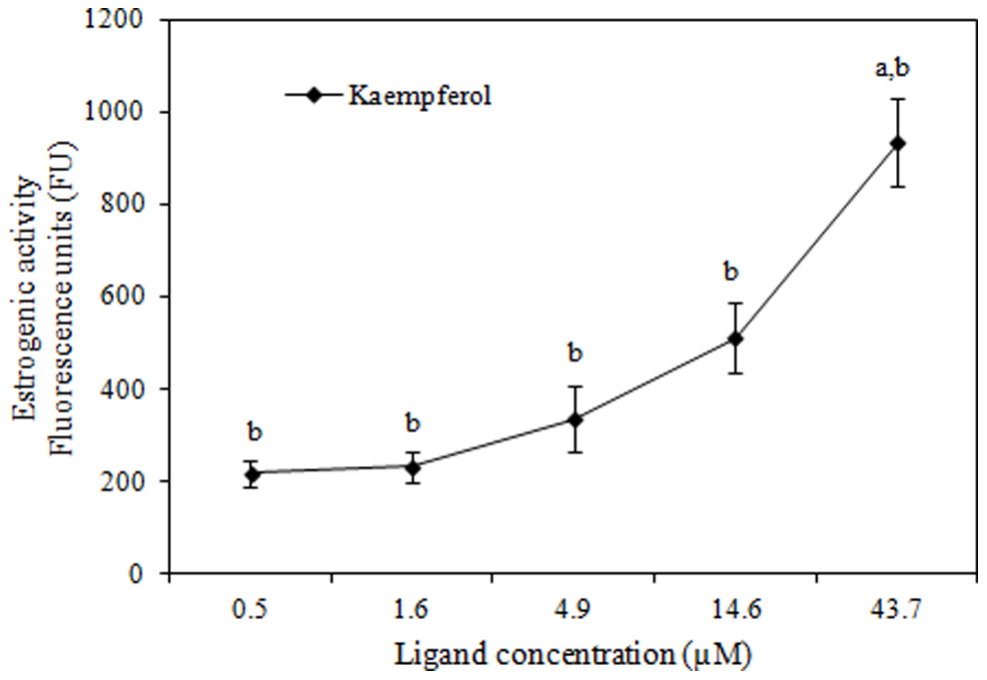
Estrogenic response for kaempferol in the recombinant yeast assay. Different concentrations of kaempferol (µM) were added to genetically engineered, estrogen- responsive yeast cells and incubated for 6 h. The β-galactosidase activities were calculated as fluorescence units (FU). Values are averages of six independent experiments; bars indicate value ranges. ^a^Significantly different from the negative control, DMSO, FU = 182±21. ^b^Significantly different from the positive control, 17-β-estradiol, FU = 9910±338 (one-way ANOVA, Tukey test; *p*≤0.05).

The variation on β-galactosidase activity at different kaempferol concentrations (tested in sextuplicate) is represented in **Figure 2**.

The effects of 17-β-estradiol and flavonoids quercetin, kaempferol, galangin, luteolin, fisetin, chrysin, 3-hydroxyflavone, 5-hydroxyflavone, 7-hydroxyflavone and flavone on MCF-7/BUS cell proliferation measured by *E-screen* are shown in **Table 2**.

**Table 2:** Effects of flavonoids (quercetin, kaempferol, galangin, luteolin, fisetin, chrysin, 3-hydroxyflavone, 5-hydroxyflavone, 7-hydroxyflavone and flavone) on MCF-7/BUS cell proliferation measured by *E-screen.*

	Quercetin		Kaempferol		Galangin		Luteolin		Fisetin	
Concentrations (M)	PE^a^	RPE^b^	PE^a^	RPE^b^	PE^a^	RPE^b^	PE^a^	RPE^b^	PE^a^	RPE^b^
c **C+**	1.41±0.36	100	1.41±0.36	100	1.41±0.36	100	1.41±0.36	100	1.41±0.36	100
**10** ^−**9**^	0.92±0.17*	−	1.17±0.48*	42.2	1.04±0.13*	9.2	1.14±0.25*	35.3	1.15±0.49*	37.2
**10** ^−**8**^	0.76±0.27*	−	1.07±0.39*	16.1	1.03±0.13*	8.3	1.13±0.29*	31.6	1.12±0.55*	29.2
**10** ^−**7**^	0.83±0.13*	−	1.11±0.48*	27.1	1.00±0.11*	0.8	1.08±0.27*	20.1	1.11±0.45*	27.0
**10** ^−**6**^	0.89±0.13*	−	1.27±0.52*	66.8	1.06±0.17*	15.0	1.09±0.25*	23.1	1.22±0.57*	53.5
**10** ^−**5**^	0.77±0.15*	−	1.35±0.26*	85.3	1.18±0.39*	44.0	1.11±0.18*	27.5	1.14±0.61*	33.3
	**Chrysin**		**3-hydroxyflavone**		**5-hydroxyflavone**		**7-hydroxyflavone**		**Flavone**	
	**PE** a	**RPE** b	**PE** a	**RPE** b	**PE** a	**RPE** b	**PE** a	**RPE** b	**PE** a	**RPE** b
c **C+**	1.41±0.36	100	1.41±0.36	100	1.41±0.36	100	1.41±0.36	100	1.41±0.36	100
**10** ^−**9**^	0.94±0.13*	−	0.91±0.11*	−	0.85±0.26*	−	0.88±0.08*	−	0.99±0.03*	−
**10** ^−**8**^	0.90±0.14*	−	0.87±0.03*	−	1.00±0.09*	−	0.93±0.06*	−	0.95±0.19*	−
**10** ^−**7**^	0.85±0.16*	−	0.79±0.04*	−	0.88±0.23*	−	0.82±0.17*	−	0.97±0.06*	−
**10** ^−**6**^	0.97±0.12*	−	0.83±0.21*	−	0.82±0.21*	−	1.00±0.02*	0.6	0.93±0.13*	−
**10** ^−**5**^	1.00±0.04*	1.0	0.56±0.22*	−	0.56±0.18*	−	1.02±0.06*	4.9	0.67±0.19*	−

a Proliferative effect (PE) is calculated as the effect on solvent control;

b Relative proliferative effect (RPE) compares the maximum proliferation induced by a sample with that induced by 17-β-estradiol;

c C+ = positive control (1×10^−8^ M 17-β-estradiol).

* Significantly different from the positive control, 17-β-estradiol (one-way ANOVA, Tukey test; *p*≤0.05).

The results are expressed in PE and RPE %. RPE is a measure of the magnitude of the response of the tested substances, relative to the reference substance 17-β-estradiol ( = 100%).

Galangin, luteolin and fisetin showed partial agonist activity, with RPE = 44.0%, 35.3% and 53.5%, respectively; however, kaempferol exhibited full agonist activity, with RPE = 85.3%, demonstrating a higher capacity to induce the MCF-7/BUS cell proliferation. Quercetin, chrysin, 3-hydroxyflavone, 5-hydroxyflavone, 7-hydroxyflavone and flavone did not induce significant cell proliferative activity (**Table 2**).

**Figure 3:**
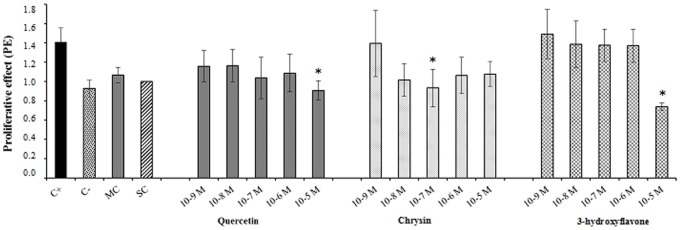
Antiestrogenic effect of various concentrations of flavonoids (quercetin, chrysin and 3-hydroxyflavone) on 17-β-estradiol-induced cell proliferation. C+ = positive control (17-β-estradiol), C− = negative control (steroid-free experimental medium), MC = medium control (10% FBS in DMEM), SC = solvent control (0.01% DMSO, the highest concentration of solvent used in the test). *Significantly different from the positive control (one-way ANOVA, Dunnett test; *p*≤0.05). Three independent experiments were done with the flavonoids, all of them in triplicate.

With respect to antiestrogenic activity, we observed that the flavonoids quercetin, chrysin and 3-hydroxyflavone significantly suppressed cell proliferation, compared to the effect of 17-β-estradiol alone, suggesting an antiestrogenic or 17-β-estradiol antagonist effect (**Figure 3**). The other compounds showed no differences in cell proliferation inhibition when compared with 17-β-estradiol alone, under the present experimental conditions (data not shown).

## Discussion

Phytoestrogens are plant-derived chemicals with estrogen-like activities, which may perform beneficial roles in human estrogen deficiency, and the most potent phytoestrogens are members of the flavonoid family [17]. These compounds have some structural similarities to the natural estrogen 17-β-estradiol, as well as to other steroid hormones and steroid hormone antagonists [18], and can interact with the ER and induce gene expression similar to that induced by estrogens, albeit at a lower affinity [19].

The ER has been shown to be able to bind to an array of compounds with a degree of structural diversity [20]. However, the potency of each substance may be due to its affinity for the ERs [21].

Given the significant interest in the estrogenic activity of phytoestrogens, this study was conducted to determine the estrogenic activity of flavonoids by the methods of *E-screen* and RYA.

By comparison with the natural estrogen, 17-β-estradiol, compounds or samples can be ranked according to their potency. Since the *in vivo* effects of estradiol are very well documented, this compound serves as a reference framework for compound prioritization or hazard identification in environmental matrices [22].

When we relate the structure of flavonoids to their estrogenic activity in the RYA and *E-screen,* it is clear that there is some flexibility in the structural characteristics necessary to induce an estrogenic response. However, the results obtained in this study indicate that subtle changes in the structure of these compounds may alter their biological activity and specificity to the ER. The number of hydroxyl groups, especially those on the B ring of the flavonoid, seems to be important, whereas changes in A- or C-ring hydroxylation are of minor importance.

According to Zand et al. [18], hydroxyl groups at position 4′ confer more potent estrogenic activity and the most potent estrogenic compounds have between 2 and 4 hydroxyl groups. At least one is bonded at position 7 of the A-ring, and another at position 4′ of the B-ring. These structural features are exhibited by the molecules of quercetin, kaempferol, fisetin and luteolin.

Interestingly the results of RYA are concordant with the data obtained in cell proliferation assay where kaempferol proved to be the only one with estrogenic activity mediated by ERα dependent transactivation, with 6.74±1.7 nM EEQ in the RYA. Furthermore, it acted as a full agonist for stimulation of proliferation of MCF-7/BUS cells. The other compounds (quercetin, fisetin, chrysin, luteolin, galangin, flavone, 3-hydroxyflavone, 5- hydroxyflavone and 7- hydroxyflavone) showed no detectable levels of estrogenicity in the conditions used in the RYA. However, in the *E-screen*, compounds such as galangin, luteolin and fisetin also stimulated proliferation of MCF-7/BUS cells, acting as partial agonists.

ER is a member of the nuclear receptor super family of proteins that modulates the expression of genes typically as a consequence of ligand binding. In addition to that some recent reports suggest that estrogens and estrogen mimicking compounds also activate diverse growth factor/mitogen-like signaling pathways, including Src/Ras/MAPK and cAMP pathways, in MCF-7 cells and these responses may be associated with activation of membrane ER [5], [23].

The discrepancy between RYA and MCF-7 test results might be due to distinct mechanisms occurring in yeast and in mammalian cells [9]. Yeast, for example, does not contain endogenous steroid or thyroid hormone receptors or related proteins such as aromatase. Moreover, the ER functions as a ligand-dependent transcription factor in the recombinant yeast. Thus, the estrogenicity of a substance can only be assessed by this mechanism in the RYA.

With respect to the antiestrogenic activity, the compounds quercetin, chrysin and 3-hydroxyflavone significantly inhibited cell proliferation induced by 17-β-estradiol in the *E-screen* assay, indicating that these compounds can act as ER antagonists. These results are highly relevant, because antiestrogens block 17-β-estradiol binding to ERα and antagonize estrogen-stimulated gene expression which is highly desirable relative to breast cancer prevention and treatment [24].

According to Oh et al. [23], antiestrogenic compounds can act by competing with 17-β-estradiol for ER binding, resulting in a functionally inactive, ligand-bound complex, or by depleting endogenous estrogen through the inhibition of estradiol biosynthesis (aromatase activity) or stimulation of estradiol catabolism. Moreover, antagonist-bound ERs adopt a distinct conformation that enables them to preferentially interact with corepressors rather than coactivators, thereby reinforcing their negative regulatory properties [24].

On the basis of structure-activity relationships analyzed by Fang et al. [25], some distinguishing features were found to be essential for xenoestrogen activity, using 17-β-estradiol as a template: (1) H-bonding capacity of the phenolic ring, mimicking the 3-OH, (2) H-bond donor mimicking the17-β-OH, (3) O-O distance between 3-and 17-β-OH, and its orientation, (4) hydrophobicity and (5) a ring structure. The elimination or modification of any of these features of the estrogen significantly reduces its binding affinity for the receptor.

Moreover, according to Anstead et al. [26], even if the overall skeletal conformation is similar, steroids lacking an aromatic ring have low binding affinity. On the other hand, according to Ward and Kuhnle [27], only an aromatic ring and a hydroxyl group are important features for a compound to bind to the receptor.

Regarding the compounds evaluated in this study, we observed that although quercetin has only one hydroxylation in the 3′-position of kaempferol [17], [28], this structure did not show estrogenic activity in either the RYA or *E-screen* assay.

This indicates that *ortho* positioning between two hydroxyl groups may serve to reduce estrogenicity [17]. Moreover, Zand et al. [18] demonstrated that a total number of groups exceeding 4 reduce the estrogenic activity of the flavonoid. Of the compounds evaluated, quercetin is the only substance that has more than four hydroxyl groups. However, quercetin proved capable of inhibiting cell proliferation induced by 17-β-estradiol significantly, demonstrating antiestrogenic activity.

The estrogenicity of fisetin and luteolin was weak, probably due to two hydroxyl groups on the B ring, which reduced the stimulation of cell proliferation and impeded direct interaction between the compounds and the ER. In the *E-screen*, these compounds exhibited some estrogenic activity, as partial agonists, while in the RYA they showed no detectable levels of estrogenicity.

Another compound that has structural properties similar to those of kaempferol is galangin, differing only in the number of hydroxyl groups on the B ring. In fact, galangin is a flavonol that does not have any hydroxyl group on the B ring [29]. In the *E-screen*, galangin also acted as a partial agonist, whereas in the RYA, the absence of hydroxyl groups on the B ring prevented interaction with the ER, demonstrating that the activity of this compound is not directly dependent on the affinity for the ER.

According to Choi et al. [17], hydroxyl groups appear to be crucial for binding activity. If hydroxyl groups are not present in the structure, no estrogenic activity is observed [18], as demonstrated in the results with flavone.

The structural modifications of the A- and C-rings do not appear to influence the estrogenic activity of these compounds, as can be observed in the results of the flavonoids 3-hydroxyflavone, 5-hydroxyflavone, 7-hydroxyflavone, flavone and chrysin in both the RYA and the *E-screen*. The lack of estrogenicity is believed to be primarily due to the absence of a hydroxyl on the B ring.

However, the presence of only one hydroxyl group in position 3, as in the molecule of 3-hydroxyflavone, and hydroxyl groups at positions 5 and 7, as in chrysin, were shown to be important in the antiestrogenic activity. These compounds inhibited significantly the cell proliferation induced by 17-β-estradiol.

Studies have suggested that the three-dimensional folding of the hormone-binding domain induced by a ligand to leads a change the ionic charge at the surface of the ligand-receptor complex. Estrogen antagonist can induce conformational modifications of the ER that do not preclude its binding to the estrogen response element but fail to promote the sequence events needed for gene transcription [30].

The double bond between carbons 2 and 3 does not seem to interfere in the estrogenicity, because all of the compounds that showed (anti) estrogenic activity have this structural feature.

However, it is well established that functional phytoestrogens belong to structurally different classes of compounds; therefore, chemical structures alone are not sufficient to predict estrogenic activity [31]. Moreover, the negative results observed in this study do not exclude the estrogenic activity of the respective tested compounds, since they may act by different mechanisms from those revealed by RYA and *E-screen*.

Further research is needed to reach a better understanding of the interactions involved in ligand-receptor binding, since a more detailed knowledge of its structure may prove valuable in the search for preventative natural phytoestrogens, as well as the development of novel drugs derived from natural products. In this context, we highlight, among the compounds tested, the estrogenic activity of kaempferol mediated by ERα-dependent transcriptional activation and by stimulation of MCF-7/BUS cell proliferation. Moreover, this work emphasizes again the need for complementary methods of analysis of estrogenic activity.
